# Method for Continuous Integration and Deployment Using a Pipeline Generator for Agile Software Projects

**DOI:** 10.3390/s22124637

**Published:** 2022-06-20

**Authors:** Ionut-Catalin Donca, Ovidiu Petru Stan, Marius Misaros, Dan Gota, Liviu Miclea

**Affiliations:** Department of Automation, Faculty of Automation and Computer Science, Technical University of Cluj-Napoca, 400114 Cluj-Napoca, Romania; ionut.donca@aut.utcluj.ro (I.-C.D.); marius.misaros@aut.utcluj.ro (M.M.); dan.gota@aut.utcluj.ro (D.G.); liviu.miclea@aut.utcluj.ro (L.M.)

**Keywords:** agile, containerization, version management, git, configuration management, continuous integration, continuous delivery

## Abstract

Lately, the software development industry is going through a slow but real transformation. Software is increasingly a part of everything, and, software developers, are trying to cope with this exploding demand through more automation. The pipelining technique of continuous integration (CI) and continuous delivery (CD) has developed considerably due to the overwhelming demand for the deployment and deliverability of new features and applications. As a result, DevOps approaches and Agile principles have been developed, in which developers collaborate closely with infrastructure engineers to guarantee that their applications are deployed quickly and reliably. Thanks to pipeline approach thinking, the efficiency of projects has greatly improved. Agile practices represent the introduction to the system of new features in each sprint delivery. Those practices may contain well-developed features or can contain bugs or failures which impact the delivery. The pipeline approach, depicted in this paper, overcomes the problems of delivery, improving the delivery timeline, the test load steps, and the benchmarking tasks. It decreases system interruption by integrating multiple test steps and adds stability and deliverability to the entire process. It provides standardization which means having an established, time-tested process to use, and can also decrease ambiguity and guesswork, guarantee quality and boost productivity. This tool is developed with an interpreted language, namely Bash, which offers an easier method to integrate it into any platform. Based on the experimental results, we demonstrate the value that this solution currently creates. This solution provides an effective and efficient way to generate, manage, customize, and automate Agile-based CI and CD projects through automated pipelines. The suggested system acts as a starting point for standard CI/CD processes, caches Docker layers for subsequent usage, and implements highly available deliverables in a Kubernetes cluster using Helm. Changing the principles of this solution and expanding it into multiple platforms (windows) will be addressed in a future discussion.

## 1. Introduction

Nowadays, conventional software development methods are insufficient for today’s business needs. Adapting to agile practices can increase the flexibility, efficiency, and speed of the software development life cycle, which is what software development companies are attracted to [1]. For this reason, many researchers and companies try to develop their own solutions to building a product that can generate and automate the entire process of Continuous Integration (CI), Continuous Delivery (CD), and Continuous Deployment (CDT) [2].

The development of a product or service is achieved through iterations or rapid development cycles carried out in a shorter time. In other words, instead of a monolithic development strategy, agile practices involve working on chunks of projects at the same time, which makes changes and adjustments easy and manageable. Initially, the developers will analyze the whole journey a functionality is expected to take: from the moment it is born, to how it is defined and prioritized over other functionalities, how the team that will implement it is chosen, how resources are allocated, and how the work is planned. Only after all these stages are complete, will the developers look at the implementation. At first, there is a dizzying amount of information and apparent chaos. However, with the process of CI/CD/CDT, the most important lean and agile principles can be followed.

CI technique has several advantages; those worth mentioning lower risk, as far as is feasible, to produce error-free and dependable software and remove limitations to the number of times an implementation may be performed. Reduced time to market, improved product quality, increased customer satisfaction, valid releases, boosted productivity, and efficiency are the main benefits that drive companies to invest in CD. Nowadays, most software and mobile development are delivered on Infrastructure as a Service (IaaS); therefore, without a doubt, CI, CD and CDT have become a major aspect of cloud computing [3].

Well-implemented systems usually pass the overall test cases, but it is difficult for them to be reverted to a previous version due to performance issues discovered after deployment or if a migration has been processed [4]. System-sizing decisions are an immediate solution to the problem, as agile processes place more emphasis on delivering products [5] within specified deadlines. Another solution is to integrate into the pipeline the rollback process, which means that if the monitoring thresholds or basic checks are violated, the system will be reverted automatically to the previous version [6].

The presented paper provides a blueprint for automated CI, CDT pipelines. It explains why well-engineered, mature delivery pipelines are important in providing both agility and quality [7].

The remainder of this paper is arranged as follows. The next section, Section 2, describes the core concepts with respect to the related work. Section 3 presents the architecture of the proposed solution. Section 4 elaborates, explains the solution with the selection of tools, and also includes chunks of code (Bash scripts). This section also outlines outlines the evaluation analysis of the proposed solution with the results and, finally, Section 5 closes this paper with conclusions and possible directions for further research or work, and future research challenges.

## 2. Literature Review

Today, the IT industry adopts CI/CDT/CD principles as one of the main technologies for application delivery and deployment to satisfy business requirements and product challenges. First, the related work that is available today on the market is described and includes a short comparison between the different studies and a proposed solution; secondly, this section describes the core concepts and principles underlying the continuous software engineering paradigm that is adopted in this proposed solution [8].

### 2.1. Related Work

#### 2.1.1. Argo CD

Like most software developed nowadays, Argo CD is an open source GitOps continuous delivery tool for Kubernetes. Argo CD automatically deploys software applications to Kubernetes, from declarative configuration files that are stored in Git. This allows the configuration to be version-controlled and auditable through Git. The tool acts as a Kubernetes controller, monitoring the state of both the source from Git and the deployed application. It searches for differences between the two of them and provides the means to update the deployed software application manually or automatically if the Git version has been updated. Beside this, it also provides a visualized report of the current state of differences via UI. Argo CD is also capable of dividing access to configuring applications through its projects that can have defined access, based on teams of developers with role-based access control. However, despite the above functions, this tool only covers the CD part of an organization, and this can be considered a minus in comparison with Ramadoni’s et al. proposed solution [9].

Argo CD cannot manage the whole software lifecycle on its own. It is appropriate for application deployment in the last stage of the delivery process, but it must be linked to another platform that can conduct all previously available procedures, such as testing and monitoring.

Academics recommend that future work should include the GitOps process [10], which involves not only development but also CI/CD and observability [11], because it is utilized as a study material. It is critical that there be solutions that can cover the complete software lifecycle [12], especially for firms that wish to implement continuously [9].

#### 2.1.2. FluxCD

FluxCD is also an open source, developed by the Cloud Native Computing Foundation. It is described as a GitOps operator for Kubernetes [10] that synchronizes the state of manifest in a Git repository to what is running in a cluster [13]. It runs in the cluster, and its principal scope is to monitor a remote Git repository to apply changes in Kubernetes manifests. This tool is easy to install and maintain because the only exclusive focus is on the deployment part of the software delivery cycle, working specifically on the synchronization of Git repositories–and container registries–with the version and state of workloads in a cluster. By comparison, this tool needs to be installed in the Kubernetes cluster, which adds workload to the management and monitorization part, whereas the solution suggested in this paper does not require this part.

The main goal of FluxCD is to keep the Kubernetes clusters in sync with configuration sources and to configure, automatically, the updates when needed (new code must be deployed). FluxCD is designed from the bottom up to make use of the Kubernetes API extension system and to interface with Prometheus and other key components of the Kubernetes ecosystem. Flux enables multi-tenancy, as well as synchronizing an unlimited number of Git repositories [13].

Beside these two applications, there are others that can be linked in some way with solution proposed in this paper. It encapsulates multiple features into one solution, so it is no longer necessary to adopt and install multiple solutions to achieve all the CI/CD principles. The proposed solution includes the following features: pipeline generator, versioning, pipeline building (CI), and pipeline deployment (CD); this paper, therefore, covers all the aspects presented in default Agile organizations.

### 2.2. Core Concepts

#### 2.2.1. Continuous Integration (CI)

A widely used software development practice is one in which developers integrate code into a shared repository multiple times a day to quickly obtain feedback on the viability of that code. CI supports automated builds and tests, so teams can quickly collaborate on a single project. Also, CI enables software companies to have a frequent and shorter release cycle [14].

This strategy facilitates a quick and reliable launch of the program in production by utilizing the available practice sets. All of this is due to the regular merging of operational software copies, which eliminates and decreases software integration difficulties and, hence, expenses. Adherents of CI urge their development teams to create software in short iterations and to merge their functional code into the root code as quickly as is feasible [15].

#### 2.2.2. Continuous Delivery (CD)

Continuous delivery is a software engineering practice in which teams design, build, test, and release software in short cycles. It relies on automation at each stage to ensure the cycle is both fast and reliable. It employs a set of practices and automatically deploys and delivers software to a production-like environment [14].

Basically, the CD is a software development strategy that automates the process by which changes made by an application developer are delivered to the code repository or the container registry, and it shows how the changes are automatically tested for errors. Thus, all the changes can be deployed in a live production environment by the operations team. By doing so, CD solves the limited visibility and communication issue between DevOps and business teams. To that end, the goal of CD is to guarantee that implementing new code requires as little work as possible [16,17,18].

#### 2.2.3. Continuous Deployment (CDT)

Continuous deployment is the process of deploying to production as soon as qualified changes in software code or architecture are ready, and without human intervention. The differences between CD and CDT can be a little tricky to distinguish [19]. So far, with CI, the application has been coded, built, and tested through an automated pipeline using code repositories and build and test systems. Once the testing is successful, the next step is to release the new version of the application. This could be by packaging the build application into an executable or an RPM package, or into an ISO of some kind and making it available online so users can download and deploy the application in their environments. Releasing the software this way, automatically through a pipeline, is known as CD, as explained in the above section. An alternative to this step would be to take the packaged application and automatically deploy it in a target environment, like an op-prem cloud solution. This automatic process in the production environment with changes in the application is referred to as *Continuous Deployment* [20,21,22]. In other words, from the beginning, when an application code is changed and pushed to the code repository, it is automatically built, tested through a build pipeline, and then released through an air release management system, and finally deployed in production to a target environment, all integrated and automated without requiring any manual intervention. In reality, applying CDT results in high-level automation [22], since each version is automatically installed in user acceptance testing or even production settings [13].

#### 2.2.4. CI/CD Pipeline

When an enterprise attempts to adopt a CI/CD pipeline, it will no longer be capable of undertaking it autonomously. First, they should practice CI to adopt CD. Whilst transferring from CI to CD, the pipeline reduces the manual execution and ultimately the complete method becomes automated. While adopting CD, all the stages are implemented through automation. CI/CD pipeline refers to planning strategy, development, and deployment [8].

#### 2.2.5. CI/CD Tools

CI/CD tools refers to applications and third-party solutions that are involved in this research. This proposed solution uses the platform, Gitlab, where the pipelines are built, developed, and generated. Also, this solution is written in Bash scripts, plain text files containing a series of commands that are normally typed in the command line [19].

GitLab is a complete DevOps platform that brings development, operations, and security teams into a single application. GitLab helps teams accelerate software delivery from weeks to minutes, while reducing development costs and security risks. The items described below constitute some of the tools provided by Gitlab and enable this solution to be easily integrated and available for any project or company [23].

##### Version Control and Repository

Version control, also known as source control, is the practice of tracking and managing changes to software code. Version control systems are software tools in which developers manage changes to source code over time. The most used and popular version controlling system with CI/CD approaches is Git. A repository is a central place in which an aggregation of data is kept and maintained in an organized way, usually in computer storage. Semantic Versioning is a widely adopted version scheme; it uses a three-part version number (Major. Minor. Patch), an optional pre-release tag, and an optional build meta tag [24].

##### Build Tools

For the Continuous Integration part, also termed the building part, this proposed solution uses Docker [25], the containerization technology, and Kaniko [26,27]. 

Typically, if a developer creates a Docker environment [28], the workflow is convoluted and most of the time relies on a trial-and-error process. Editing a specification (e.g., a Docker file), building it into an image, instantiating the image in a container, understanding whether it works as expected or not, identifying the cause(s) of a fault, and returning to the first step to improve the specification are all steps in creating a Docker container. However, Docker provides features such as caching, customizing, scaling, and security, and works cross-platform. Another advantage of Docker, as shown by Bhimani et al. [29], is that by using specialized controls, it is possible to decide on several Docker containers that can run concurrently to reduce the overall execution time and avoid interruptions to work tasks. Kaniko is a tool to build container images from a Docker file, inside a container or Kubernetes cluster. Practically, this step prepares, wraps, and pushes the application to go to the next step [30,31].

##### Automation Tests

Test automation orchestration includes unit, functional, and performance test phases. The continuous testing approach has the benefit of a stable code base, faster response, and easy decision-making. Automation tests are an optional feature in the proposed solution [32].

##### Deployment Tools

For the Continuous Delivery and Deployment part, this solution uses Kubernetes, and more precisely, Helm [33]. Kubernetes is an open-source system for automating the deployment, scaling, and management of containerized applications. Helm is the first application package manager running atop Kubernetes [34]. It allows describing of the application structure through convenient helm-charts and managing it with simple commands.

## 3. Proposed Solution Architecture

**Figure 1:**
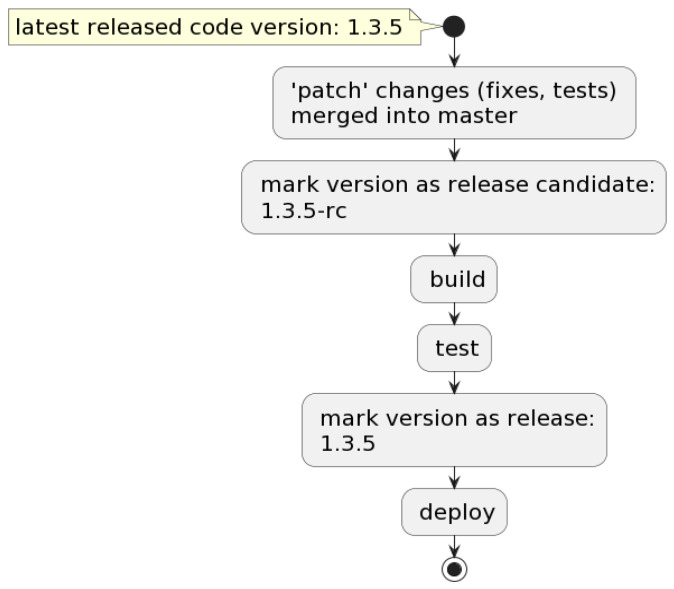
UML Diagram of the proposed solution.

In Figure 1, is pictured the simple UML Diagram of the proposed solution, with the main steps of the pipeline.

**Figure 2:**
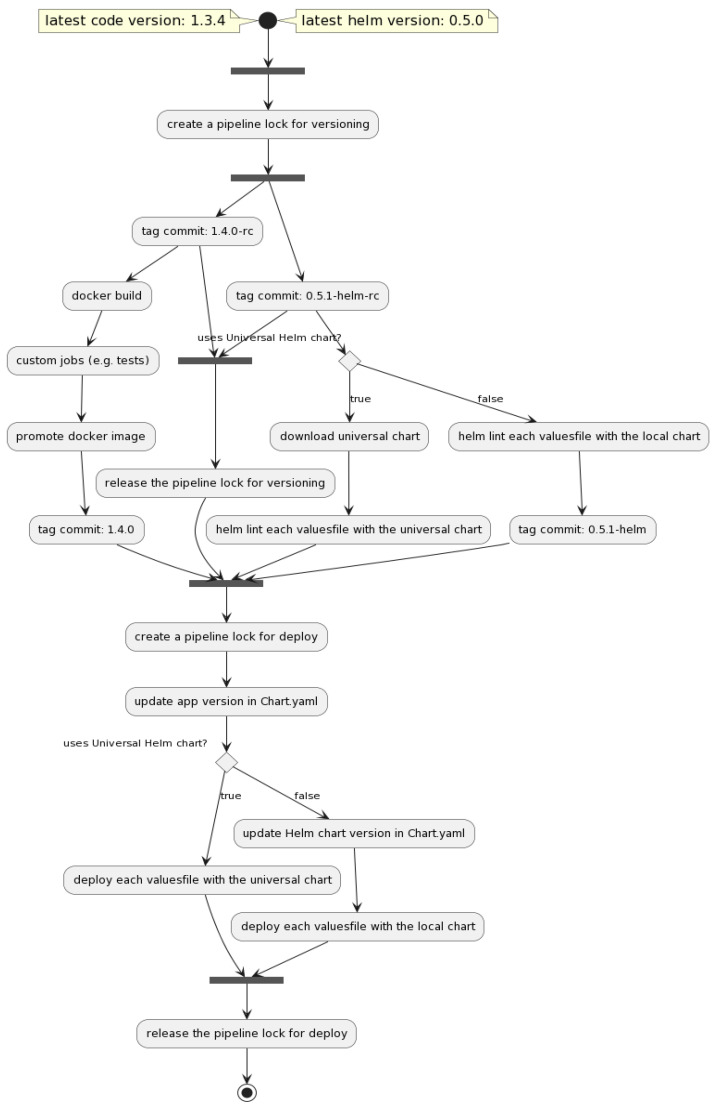
Extended UML Diagram of the proposed solution.

Figure 2 offers a better and more detailed overview of the UML Diagram of the pipeline generator and Table 1 presents the supported flows.

The following presents the entire proposed solution flows, by describing and explaining them individually and comprehensively. The proposed application is written in Bash scripting language, by one script per flow.

### 3.1. Pipeline Generator

The pipeline generator discovers build and deployment files in the git repositories and automatically creates pipeline jobs to execute them. To ensure that pipelines that inherit the CI/CD templates are always up to date, the pipeline will auto-regenerate on merge requests.

The generator creates an extra job that executes the regenerate pipeline script:

- the pipeline configuration files (i.e., *.gitlab-ci.yml* and the files from the *.ci/ folder*) are regenerated with the latest stable templates;
- if there are changes, the pipeline configuration files are pushed back to the remote HEAD, and the current pipeline is forced to terminate.

A new pipeline is triggered each time the pipeline configuration file is changed, but only for Merge Requests. Any changes made to the *.gitlab-ci.yml* or files from the *.ci/* folder other than those from the pipeline generator are ephemeral. Every time a Merge Request pipeline has been executed any changes to it will be lost. The only exception is the *.ci/custom.yml* file, which can contain any custom jobs, and which is persistent between pipeline regeneration.

The pipeline generator has two ways to regenerate: one if it is used with the default template jobs and the if it is used with custom jobs. The default jobs are basically jobs that are executed on most of the git repositories: code versioning, creating an AWS ECR repository, building Docker images, and deploying Helm charts on Kubernetes. These types of jobs are the basis of any pipeline and provide a centralized way to version, build, and deploy the code. While projects have much in common, there are still actions that are specific to each environment. Some projects may require uploading a file to an AWS S3 bucket, while others may need to run an extended test suite. To ensure the flexibility of this framework, the ability to extend the default pipeline was added. To include custom jobs in the pipeline, the need to add them to a file called *.ci/custom.yml* arises.

**Figure 3:**
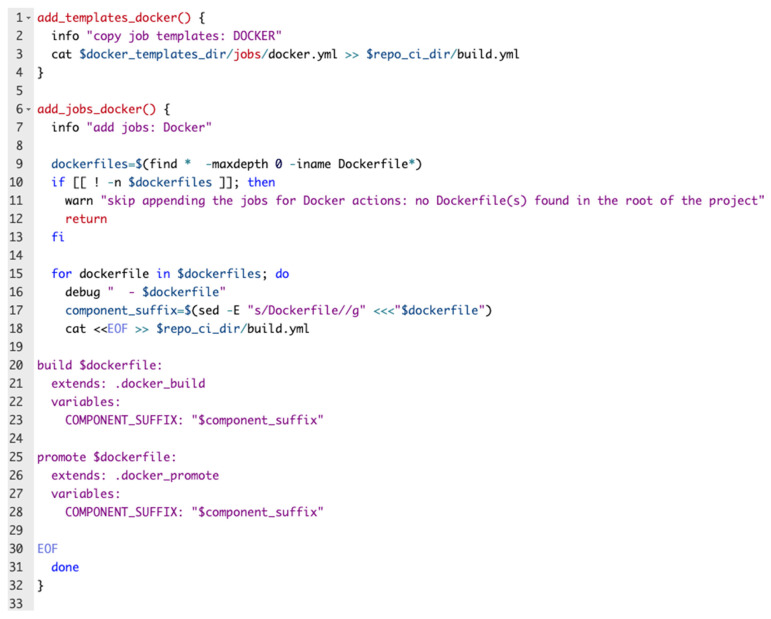
Pipeline generator code snippet.

A code snippet from the Bash script used for regenerating the pipeline is depicted in the following figure (Figure 3):

### 3.2. Versioning

**Figure 4:**
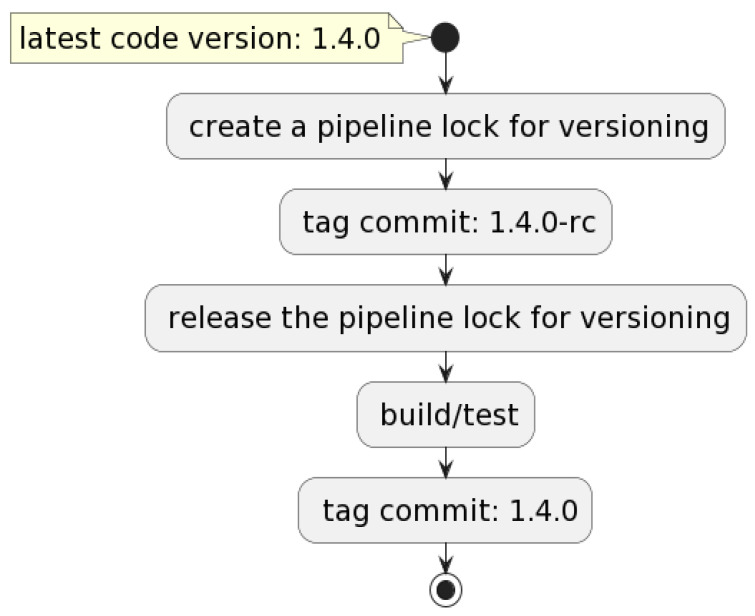
Versioning flow described.

**Figure 5:**
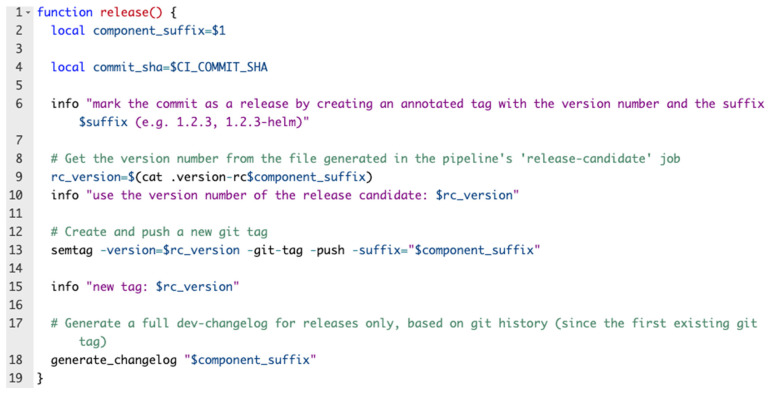
Versioning code block.

This flow is performed first and is triggered when a git commit is merged into the master: the pipeline creates a lock for versioning so that other pipelines will not start until versioning is complete. If the commit contains deployable code changes, the commit is marked as a release candidate, then the pipeline versioning lock is released. Further, the test suites are executed, and if all the tests have passed, then the commit is marked as a stable release. Figure 4 exemplifies the entire flow, while Figure 5 represents a code block from the versioning Bash script.

### 3.3. Build

In the following, the CI principles in just one pipeline are detailed. If there are any buildable code changes, the pipeline triggers a job that builds an image from the Docker file, and the build context as the root of the project. The image is tagged with two tags: a stable tag (the prefix—e.g., *rc*—and the branch name—e.g., *rc-master*), and a unique tag (the prefix—e.g., *rc*—and the branch name and the commit hash—e.g., *rc-master-adc11dad*). The image labels are updated in this way: the generic labels are appended to the image labels and, afterward, the image is published to the Container Repository: the AWS Elastic Container Registry. This flow expects to have one or more Docker files in the repository and will do the same steps for all of them.

An image is promoted only on the master branch when there are deployable code changes, by adding the semantic version tags to the latest image built on this branch and pushing the image to the Container Repository, which can be from different providers.

**Figure 6:**
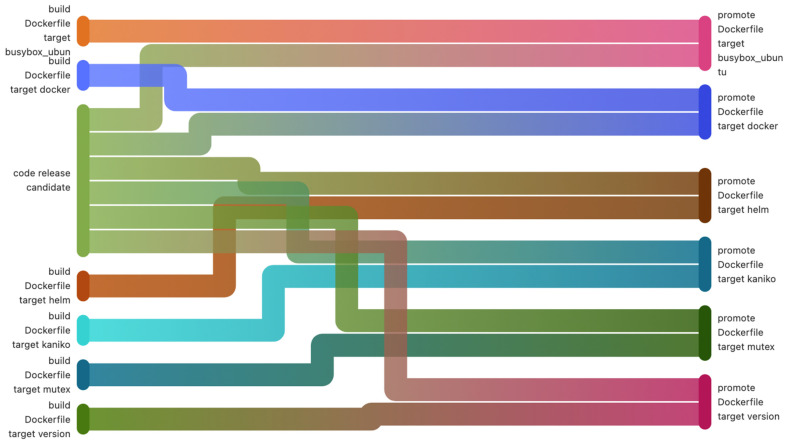
Pipeline diagram flow.

**Figure 7:**
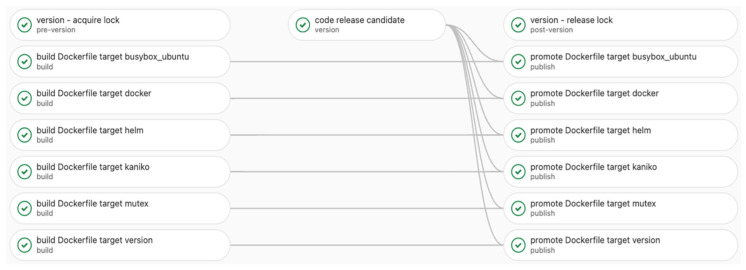
The pipeline diagram flow dependencies.

In Figure 6 and Figure 7, below, an entire build step with its dependencies is exemplified. The first figure (Figure 6) is a pipeline diagram provided by GitLab, which describes more clearly the processes that are part of the pipeline. Figure 7 shows an alternative way of describing the pipeline by also showing the jobs’ dependencies.

**Figure 8:**
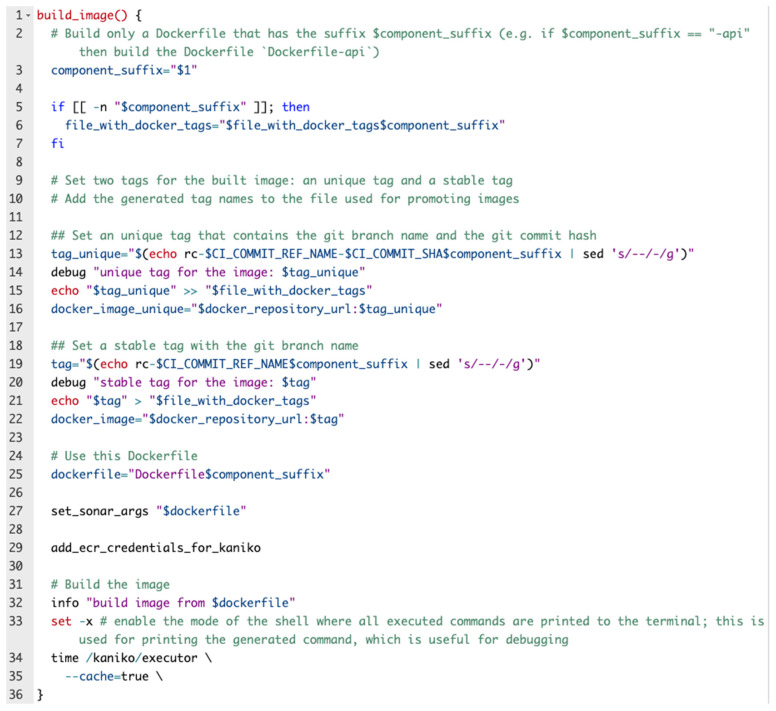
Build image function.

Figure 8 depicts one function that is part of the build Bash script which completes the CI part of this solution.

**Figure 9:**
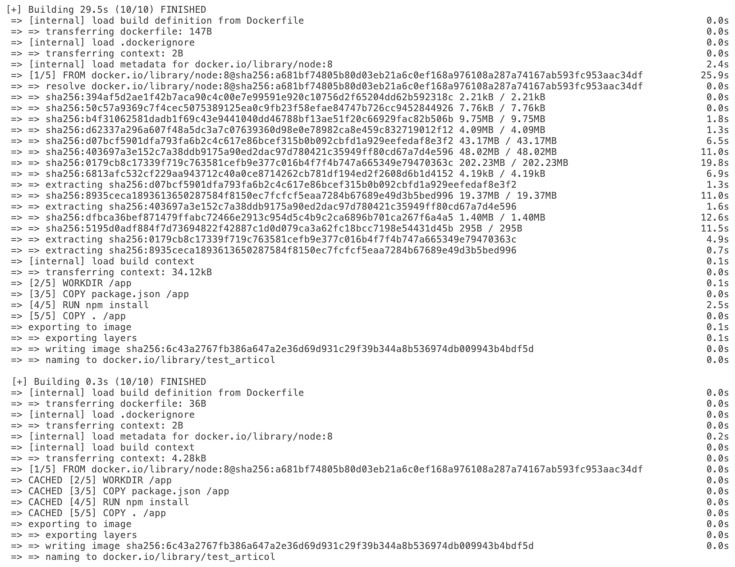
Docker-cached layers dataset.

In this subsection, another feature regarding Docker images is detailed, specifically, the Docker caching layers. Docker images are made of numerous layers, each one of which contains a set of instructions and actions defined in the Dockerfile. Layers permit Docker to isolate a large task into smaller ones, to such an extent that when a piece of the code or program is changed, only the corresponding layer of the modification should be changed. The proposed solution is very focused on caching all the layers in order to improve the speed of the build pipeline and searching for the particularly uncached layers to cache them. Figure 9 contains a detailed view of how Docker caching works and provides related time datasets, used to build the same image, first without cache and second with cache.

### 3.4. Deploy

This subsection covers the CD principles in one pipeline. It verifies whether there are any object definitions or values files. The deployment pipeline is automatically triggered if it detects any such files. This pipeline contains the linting step, which is executed to test the chart with a specific values file, and contains the version update step, which updates the application and chart versions in *Chart.yaml* file.

If the deployment step fails, a rollback job is triggered to bring the application back to the stable variant.

This solution can be easily adapted to any type of preference or any type of customization. It is easy to use it because the developed code can be easily integrated with multiple third parties.

**Figure 10:**
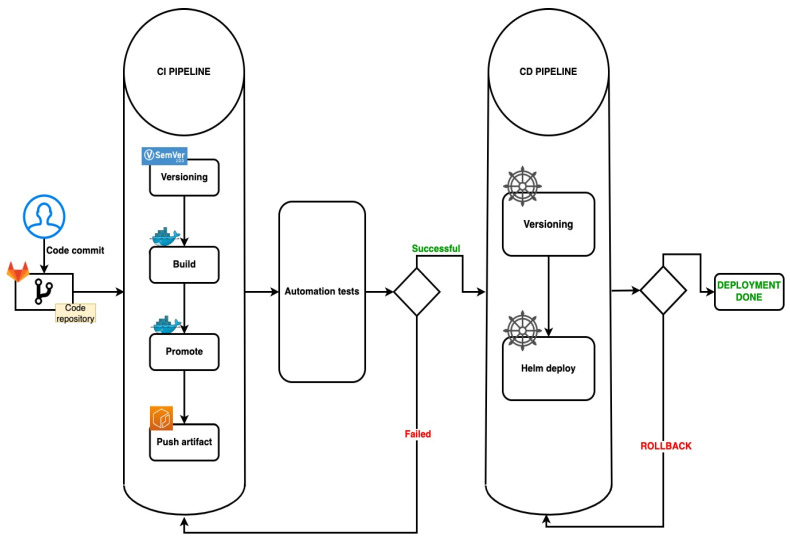
An overall proposed solution flowchart diagram.

Figure 10 represents a diagram flow, presenting the entire automated pipeline which was described in this section.

**Figure 11:**
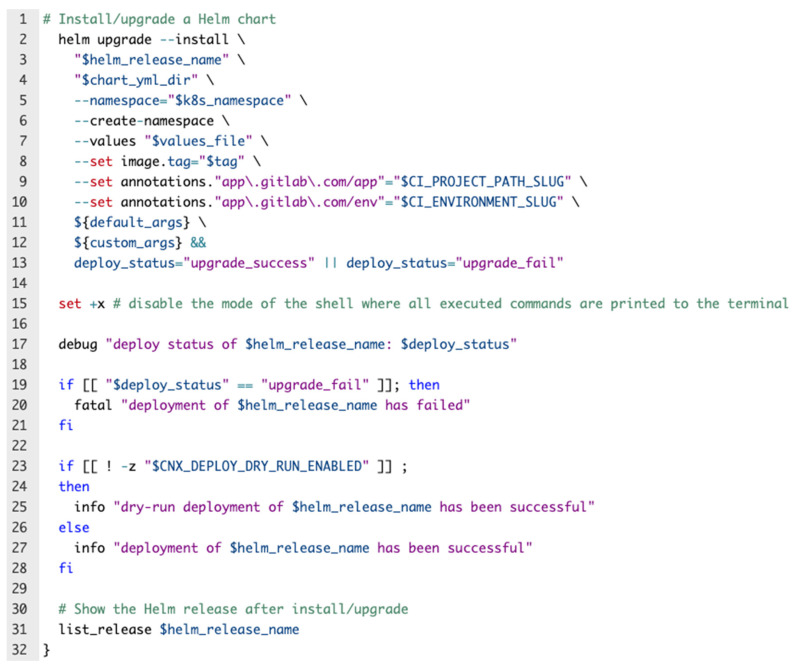
Helm installation/upgrade process.

The helm installation/upgrade process encapsulated in the deploy pipeline of the proposed solution can be easily observed in Figure 11.

## 4. Application Effect Analysis and Experimental Results

### 4.1. Application Effect Analysis

Firstly, the implementation of this solution to a project that would, otherwise, build manually its pipelines has a great impact on time-consuming deliverability, because all the steps of a complete pipeline are generated immediately and automatically, deploying an application from scratch in 10 min [35].

Secondly, the pipelines are well structured and written so that code duplication would be minimal across repositories; this means that each pipeline is unique and minimal, with nothing unused or added.

Thirdly, it reduces the financial costs because the developers and infrastructure engineers (DevOps) can focus only on their day-to-day, development and optimization tasks; they do not need to be involved in creating, managing, repairing, or monitoring the pipelines [36].

Fourthly, decreasing the ambiguity regarding the CI/CD principles creates more standardization and quality at the processes level in a project or company.

At the same time, the continuous integration deployment system is used to standardize the information system of the continuous integration release process, which greatly improves the recovery efficiency and effectively reduces the number of people involved in the development, as well as the time for personnel intervention. Moreover, the development team relieves the operating pressure, and the operation mode changes from large-scale management to refined and intensive management [37]. Management transformation improves the comprehensive quality of information systems [36]. The professional ability of operation and maintenance personnel has improved. The technology-driven transformation of operation and maintenance management has achieved great success. Data and practice show that the introduction of the continuous integration delivery system gradually improved the reconstruction and development of the operation and maintenance system [38].

### 4.2. Experimental Results

This section describes and presents the results obtained using the proposed solution in every step of the pipeline. The GitLab runners are the abstract objects responsible for executing one or more pipeline jobs. These runners are hosted in a Kubernetes environment built on the Elastic Kubernetes Service (EKS) in Amazon Web Services (AWS).

The first conclusion one can draw on the obtained result is that the runners are reusable and run-on SPOT instances, which represent AWS’s excess capacity, meaning that the price of those instances are very low compared with on-demand prices. This proposed solution feature manages to save around 80% of the computed costs on each triggered pipeline. 

Another point worth mentioning is the advantage that comes from the proposed solution, and which is related to the build step of the pipeline. Docker-cache-layer integration is a convenient way of speeding up the build process and transfers less data by reusing existing layers when possible. This means that each subsequent build will be cached and the next time a full build step is triggered, it will automatically use the cached layers. This entire process boost the pipeline’ duration, thereby the costs are minimized and the impact on the productivity side is tweaked. 

The experimental results come from the comparison of three pipeline types: the first was automated and managed through this proposed solution; the second was undertaken manually, with each step being run separately and individually; and the third was implemented through the Gitlab CI/CD solution, which does not have all the features of the proposed solution, but in which the pipelines are triggered automatically. This comparison was made to show the impact of the solution in an organization which undertakes the CI/CD processes manually, or in an organization that has started using the automation of CI/CD pipelines but is missing some important features. 

**Figure 12:**
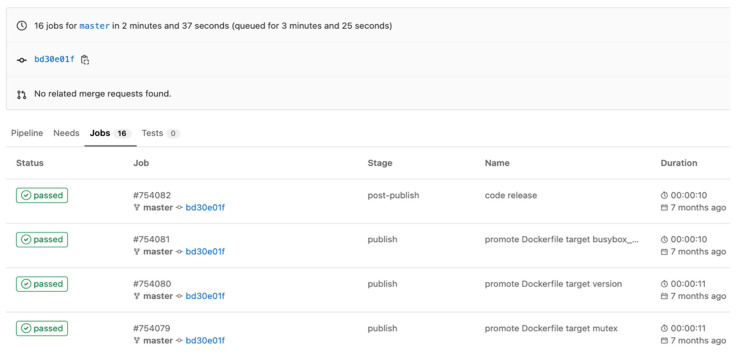
Pipeline steps and duration.

As one can observe in Figure 12, the duration of jobs from the exemplified pipeline described in Figure 6 and Figure 7 is listed. It shows that the entire pipeline takes 157 s to complete 16 jobs (6 Docker builds, 6 Docker promote, and 4 versioning jobs).

**Figure 13:**
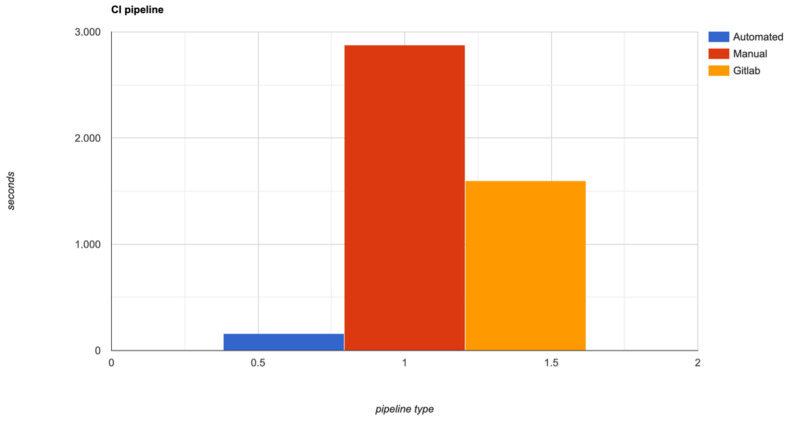
Pipeline type time difference in seconds.

The jobs were also triggered manually, without the integration of the proposed solution and it took around 180 s per each job. We have to specify that in this experiment the time consumption of triggering the jobs were not considered. If one wants to take this time into consideration, than the time execution of the the manual jobs is around 2880 s. The Gitlab CI/CD solution also automatically triggered the above jobs, but not having the caching layer and not having the relations between steps, it increased the time and the costs. For the third pipeline type, it took around 100 s per each job, with a total duration of 1600 s. Figure 13 demonstrates the difference in time between the three types of pipelines.

**Figure 14:**
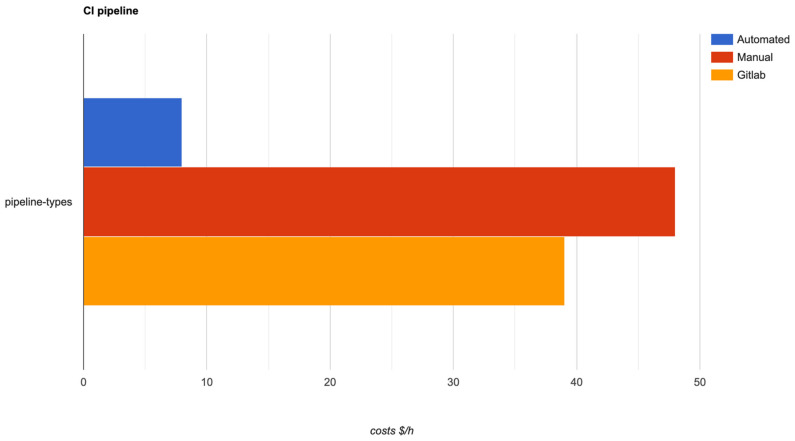
Pipeline type infrastructure costs.

The following short discussion resulting from the comparison is related with the costs for running the pipelines. With the proposed solution, the costs are as minimal as they can be, because the instances used are spot and the applications (runners) which are running the pipeline jobs, are reusable and configured in such a way as to run multiple jobs in parallel. Taken together, these can help organizations save around 80–85% of their CI/CD infrastructure costs, compared with the manual pipeline. The third type of pipeline is also able to run jobs in parallel, but it is not able to run on spot instances, meaning that the saving percentage is much less—around 10–15%—than that of the proposed solution. The results of this comparison are shown in Figure 14, where the same class of instance, with the same CPU and RAM, was used.

The final result that adds value to this proposed solution is the deployment step, which is implemented via Helm. This improves the duration of the automated pipeline because it acts like a wrapper and removes the microservices complexity that is added by managing configuration and manifest files. It provides the ability to leverage Kubernetes packages through a single command, offers the ability to customize application configurations during the deployment step, and automatically maintains a database of all the versions of deployments, so, rolling back to the previous version is easy and quick. Taking all these into consideration, the deployment step is safe, secure, and fast.

The previous section detailed parts of the Docker-cached layers which are linked with the build step that is part of the continuous integration pipeline; the results of an entire flow of this proposed solution are, therefore, depicted next. 

**Figure 15:**
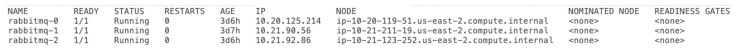
RabbitMQ deployed with the proposed solution.

These results are based on the hardware implementation that this solution requires to demonstrate its advantages. The entire solution was deployed onto the Elastic Kubernetes Services platform, which is a service provided by Amazon Web Services, on cloud instances (EC2). Once the integration was achieved, an entire pipeline of the developed solution was triggered in order to deploy a three pods RabbitMQ Helm Chart: one main and two workers. This process took around 10 min because once the pipeline started, the virtual machines needed to be assigned to the Kubernetes cluster. After this, the runners started executing the instructions defined in the pipeline manifests and finished the steps with a highly available and scalable RabbitMQ cluster. Figure 15 depicts the pods deployed on EC2 instances, each being deployed on a separate instance to avoid the probability of interruptions. 

## 5. Conclusions

Continuous integration systems help project members focus various resources on key issues, thereby reducing development time and improving software quality. The development team can spend more resources on software design; the double integration work is undertaken by machine. Continuous rapid feedback enables testers to be adequately tested. The continuous integration delivery system is a breakthrough for automated operation and maintenance. It helps to improve the maturity of software projects, implement continuous improvement of lean processes, promote the improvement of software service levels, and promote high-quality development of software systems through operation and maintenance mechanisms.

This paper presents a complex and automated pipeline generator with CI/CD principles for the deployment of multiple types of applications. The solution is based on Agile practices, which are responsible for the automatic integration, testing, and delivery of features for applications.

The proposed solution serves as a baseline for common CI/CD tasks and encapsulates the following specifics: all code must be versioned by semantic versioning standards; builds are created automatically by using Docker and the Docker layers are cached for later reuse; most deliverables are submitted to the Docker Container Registry; and most deliverables are deployed to a Kubernetes cluster via Helm. These practices ensure high availability with no downtime, fast and easy scalability, rolling back automatically to a stable version, scanning vulnerabilities in Docker, detecting any change in the application source code, and triggering an entire chain of actions and events based on what has been changed. If there are changes on the infrastructure manifests, but not on the application code, the process of building and testing pipelines will not be triggered, thereby the same artifacts’ “pollution” is bring down. This feature, even if the same artifacts has disctinct tag, leads to higher speed on pipeline duration.

### Discussion, Limitations and Future Work

Continuous integration and continuous deployment increased the speed of delivering new features and implementations into applications and radically changed the approach on how entities or businesses release and upgrade their products online [31]. With the power of cloud platforms, those integrations are built and released quite easily without downtimes and bottlenecks [39], which places this solution in the column of tools with few limitations on their side [40].

Given that this proposed solution was written in Bash scripts, the first improvement could be to rewrite it in Golang [41], because this would turn it into a cross-platform solution [42]. Also, Golang would accelerate the compilation and developments steps [43].

Another future work would be to have the ability to build and deploy applications on multiple platforms, e.g., Windows, Arm. This feature would cover the user’s needs from a platform perspective. Basically, by deploying the same solution across many public and private clouds, an algorithm (controller) that determines the optimum cloud to run the application on in terms of latency could be developed.

This proposed solution has some future research challenges as it distinguished the difficulties that might occur because of CI/CD/CDD practices. One challenge is related to how organizations will implement the continuous practices, and how context, perception, and limitations might impact CI/CD/CDD practice implementation. For instance, organizational context impacts how they do or do not maintain a repository. In spite of the fact that some of the organizations may want to suppress merge conflicts, there are other drivers that influence their decision in this regard. How each organization enables testing also varies, depending on testing impact on build times and a need to do manual or automation testing. Similarly, maintaining a fast build may seem like an ideal goal, but it is not always possible. In conclusion, automated deployment may be influenced by security needs and deployment privileges because organizations perceive security to be more important than frequent deployment.

## Figures and Tables

**Table 1:** Proposed solution necessary steps.

Nr.crt.	Step	Observation
1	(Re)generate pipeline	It discovers build and deployment files in the git repositories
2	Create Amazon (AMW) Elastic Container Repository (ECR)	
3	Versioning with annotated git tags	Used for App code Used for Helm code
4	Containerization with Docker	Docker build Docker push Promote a Docker image
5	Deployment with Helm	Helm Chart Template Helm lint App version update in Helm’s Chart.yaml file Helm upgrade
